# Anesthetic management of a patient with a continuous-flow left ventricular assist device for video-assisted thoracoscopic surgery: a case report

**DOI:** 10.1186/s12871-020-0933-1

**Published:** 2020-01-20

**Authors:** Shihoko Iwata, Sumire Yokokawa, Mihoshi Sato, Makoto Ozaki

**Affiliations:** Department of Anesthesiology, Tokyo Women’s Medical University Hospital, 8-1 Kawada-cho, Shinjuku-ku, Tokyo, 162-8666 Japan

**Keywords:** One-lung ventilation, Left ventricular assist device, Transesophageal echocardiography, Pneumothorax, Video-assisted thoracoscopic surgery

## Abstract

**Background:**

As patients with left ventricular assist device (LVAD) have long expected survival, the incidence of noncardiac surgery in this patient population is increasing. Here, we present the anesthetic management of a patient with a continuous-flow LVAD who underwent video-assisted thoracic surgery (VATS).

**Case presentation:**

A 37-year-old man with LVAD was scheduled to undergo VATS because of repeated spontaneous pneumothorax. Generally, patients with these devices have marginal right heart function; therefore, it is important to avoid factors that worsen pulmonary vascular resistance (PVR). However, VATS requires one-lung ventilation (OLV) and it tends to cause increase in PVR, leading to right heart failure.

In the present case, when the patient was set in a lateral decubitus position and progressive hypoxia was observed during OLV, transesophageal echocardiography demonstrated a dilated right ventricle and a temporally flattened interventricular septum, and the central venous pressure increased to approximately 20 mmHg. Because we anticipated deterioration of right heart function, dobutamine and milrinone were administered and/or respirator settings were changed to decrease PVR for maintaining LVAD performance. Finally, resection of a bulla was completed, and the patient was discharged in stable condition on postoperative day 37.

**Conclusions:**

The anesthetic management of a patient with LVAD during VATS is challenging because the possible hemodynamic changes induced by hypoxia associated with OLV affect LVAD performance and right heart function. In our experience, VATS that requires OLV will be well tolerated in a patient with LVAD with preserved right heart function, and a multidisciplinary approach to maintain right heart function will be needed.

## Background

Left ventricular assist devices (LVADs) are gaining popularity as a viable treatment, and patients with LVADs survive for many years; consequently, such patients who require noncardiac surgery are becoming increasingly common [1, 2]. The management of LVAD-supported patients for noncardiac surgery presents many challenges, and case reports of patients with LVADs who underwent several different types of noncardiac surgeries have been published [3–10]. However, none of these cases reported the perioperative management of video-assisted thoracic surgery (VATS). Here, we present the case of a patient with a continuous-flow LVAD in whom VATS and resection of a bulla were successfully completed.

## Case presentation

**Fig. 1:**
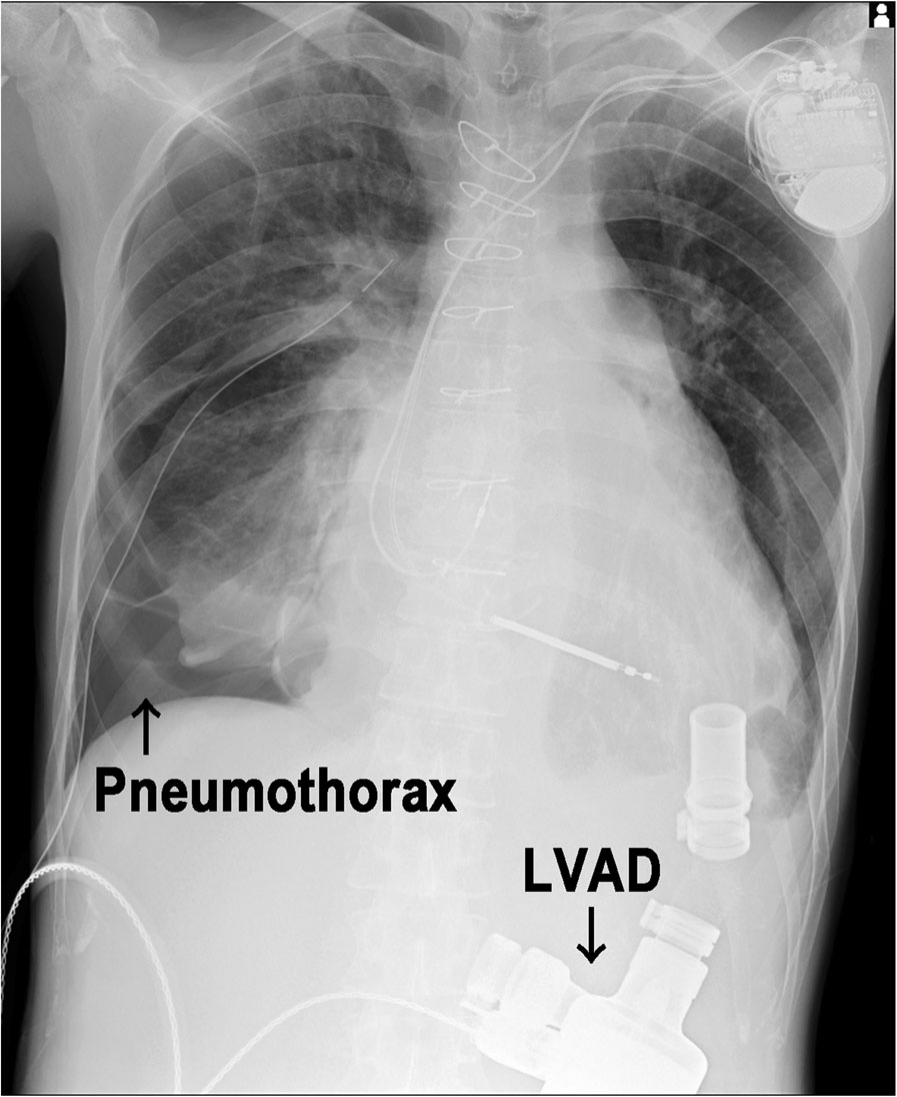
Chest radiograph. Chest radiograph showing the right-side pneumothorax with a chest tube. LVAD: left ventricular assist device

**Fig. 2:**
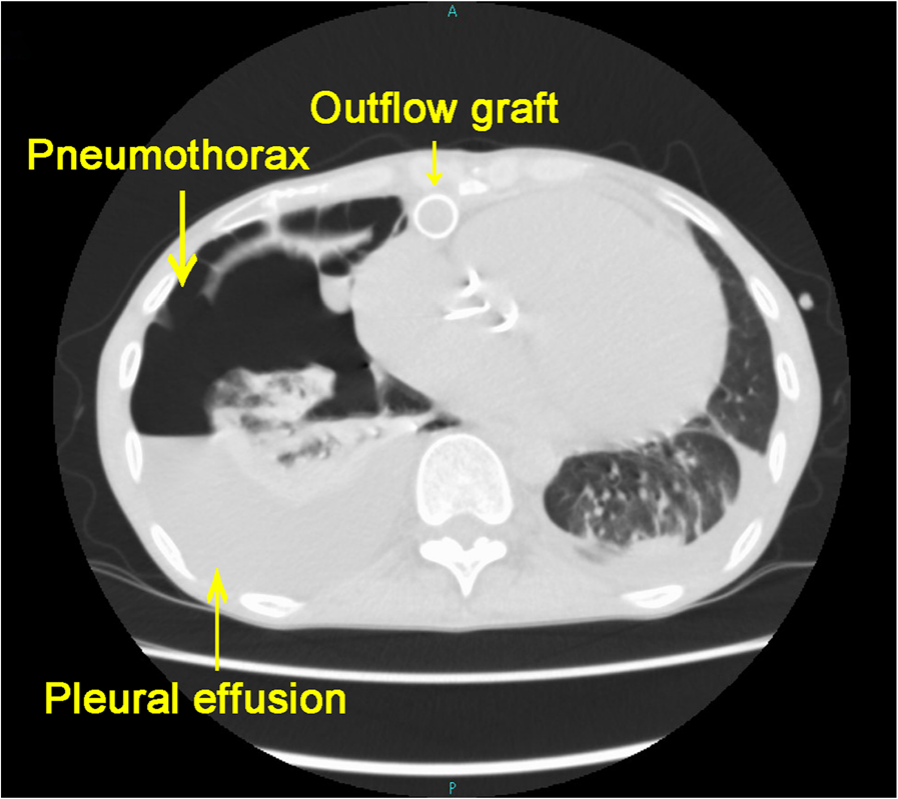
A computed tomography image of the chest in lung window. Computed tomography showing the right-side pneumothorax with moderately retained pleural effusion

A 37-year-old man (weight 61 kg, height 183 cm) was diagnosed with Becker muscular dystrophy-associated cardiomyopathy. Eventually, he received implantation of EVAHEART® (Sun Medical Technology Research Corporation, Nagano, Japan), which is an implantable centrifugal LVAD, in addition to tricuspid annuloplasty (TAP) and patent foramen ovale closure surgery. One month later and 2 months after LVAD implantation, he developed spontaneous right-sided pneumothorax. Chest radiography and computed tomography (CT) revealed right-sided pneumothorax (Figs. 1 and 2, respectively). Moreover, bullae of the right pulmonary apex with moderately retained pleural effusion were observed on CT images. Subsequently, the patient was scheduled for VATS.

Prior to surgery, the patient’s body weight decreased due to loss of appetite. He was hydrated with crystalloids at the request of the cardiac surgery team to prevent occurrence of the sucking phenomenon and the formation of thrombi. Consequently, he gained 3.5 kg of body weight in 4 days, exceeding his target body weight by 1 kg. However, a preoperative chest radiograph showed a dilated cardiac shadow and pulmonary congestion. The patient was routinely administered aspirin (100 mg) each day and warfarin to maintain the prothrombin time-international normalized ratio (PT-INR) at approximately 2.5–3.5 for systemic anticoagulation. The patient bridged from warfarin to intravenous heparin 3 days preoperatively. On the day of the operation, heparin administration was discontinued 3 h before surgery. Routine laboratory test results were within the normal limits except for anemia and coagulation abnormalities: prothrombin time: 17 s, activated partial thromboplastin time (APTT): 52.9 s (APTT control: 32.9 s), and PT-INR: 1.51.

The patient was transported to the operating room, and monitors were placed according to the Standard American Society of Anesthesiologists. An arterial catheter was inserted in the left radial artery before general anesthesia induction using an ultrasound-guided technique. The pump speed was set at 1700 rpm, pump power consumption was 2.8 W, and the mean arterial blood pressure (ABP) was approximately 80 mmHg.

**Fig. 3:**
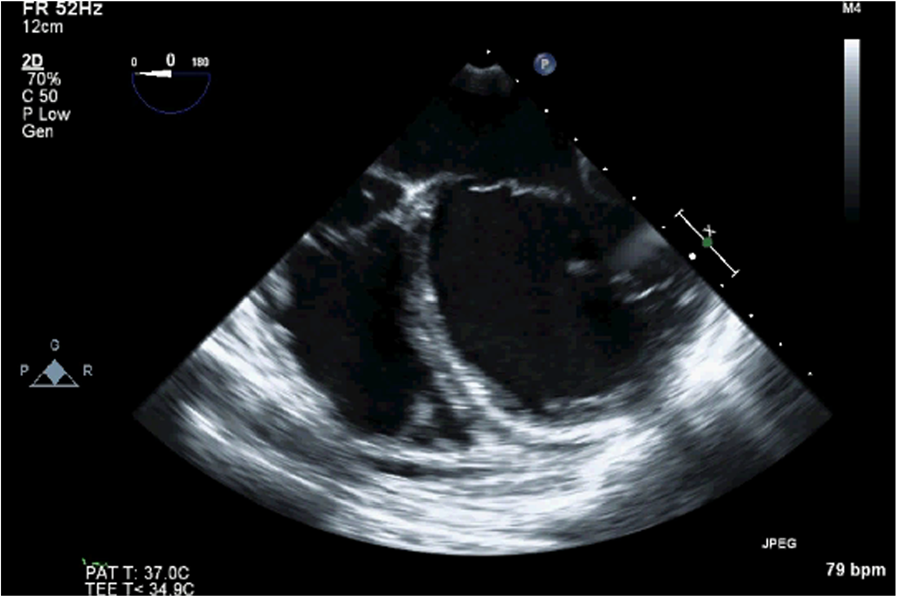
A transesophageal echocardiographic image. Mid-esophageal 4-chamber view showing the severely dilated right and left ventricles. The intraventricular septum was bowing into the right ventricle

Anesthetic induction was achieved with midazolam 2 mg, remifentanil 0.2 μg/kg/min, and a propofol target blood concentration at 2 μg/ml using the target-controlled infusion system, with maintenance doses of remifentanil at 0.2–0.5 μg/kg/min and propofol at 2–2.5 μg/ml. To facilitate endotracheal intubation using a left-sided double lumen tube Broncho-Cath™ (Mallinckrodt Medical, Athlone, Ireland), 50 mg of rocuronium bromide was administered. We did not administer additional muscle relaxants except when a 10 mg maintenance dose of rocuronium bromide was used just after the patient was placed into the left lateral decubitus position, and 20 mg rocuronium bromide was administered just before the first incision. The train-of-four was measured for neuromuscular monitoring throughout the operation. A central line was inserted in the right jugular vein. The patient’s ABP and central venous pressure (CVP) were continuously monitored using invasive means. Electrocardiography showed the sinus rhythm supported by the pacemaker, but the mode was changed from DDD to DOO at the heart rate of 80 bpm to avoid interference of electrocautery. Defibrillator pads were attached because implantable cardioverter defibrillator detection was inactivated. A transesophageal echocardiography (TEE) probe was placed for monitoring the performance of the LVAD and to determine intraoperative cardiac function. Initial findings included severely dilated left ventricle (LV) and right ventricle (RV), bowing of the intraventricular septum into the RV (Fig. 3), reduced LV and RV wall motion [RV-fractional area change (FAC) 16.8%], severe mitral regurgitation, continuous closed aortic valves, no aortic regurgitation, and trivial tricuspid regurgitation (TR) after TAP. The inflow cannula, anastomosed to the apex of the LV, and the outflow cannula, attached to the ascending aorta, of the LVAD were unobstructed with low flow velocities of 90.2 cm/s and 87.3 cm/s on pulse wave and color Doppler analyses, respectively. The right ventricular systolic pressure (RVSP) was estimated at 21 mmHg by measuring the TR jet maximum velocity. First, arterial gas analysis showed the following results: pH 7.437, pCO_2_ 31.1 mmHg, pO_2_ 225 mmHg, BEecf 2.7, lactate 0.7 mmol/L, SaO_2_ 99% and Hb 9.2 g/dL at FiO_2_ of 0.5.

**Fig. 4:**
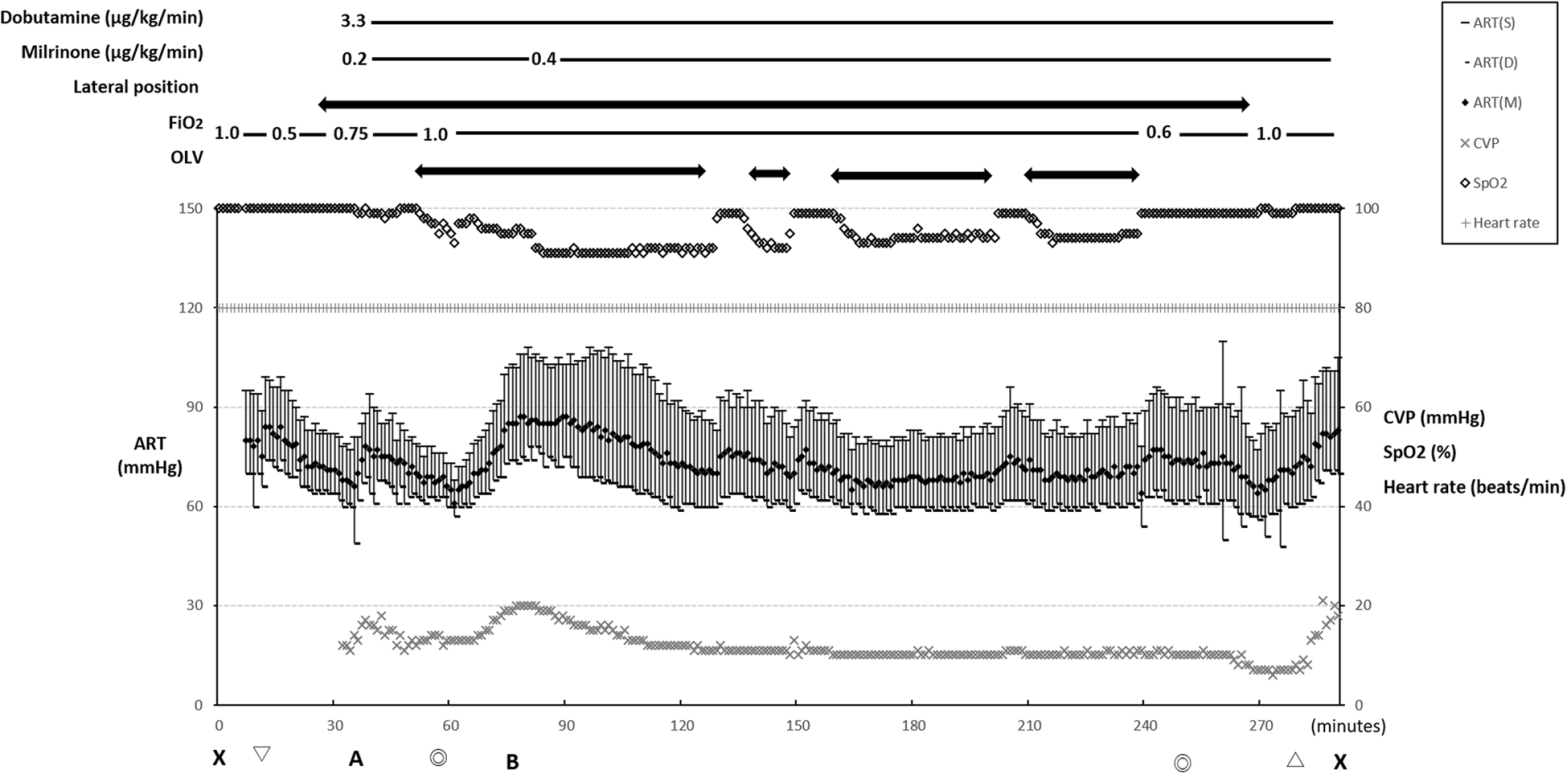
Anesthetic record. The pulsatile waveforms were maintained on ABP. When the patient was placed in the left lateral decubitus position, CVP increased from 12 mmHg to 20 mmHg (A). After insertion of the access ports, SpO2 decreased to 91% at FiO2 of 1.0, and the CVP reached 20 mmHg, although the mean ABP was maintained at approximately 85 mmHg (B). ABP: arterial blood pressure, CVP: central venous pressure, SpO2: saturation of percutaneous oxygen, FiO2: fraction of inspiratory oxygen, OLV: one-lung ventilation. X: start and completion of anesthesia, ◎: start and completion of surgery, ▽: intubation, △: extubation

When the patient was placed in the left lateral decubitus position, the CVP increased from 12 mmHg to 20 mmHg despite maintaining a mean ABP of 80 mmHg (Fig. 4 (A)). The external portion of driveline was not kinked. As the patient was coughing slightly, 10 mg rocuronium bromide was administered, and the dose of remifentanil was increased. TEE examination revealed an almost akinetic LV, free wall RV, and a flattened intraventricular septum. The inflow and outflow cannulas of the LVAD were noted to be in the proper position with a pulsatile flow of approximately 60 cm/s and 110 cm/s, respectively. We assessed that the patient needed inotropic support, and hence, the administration of dobutamine (3.3 μg/kg/min) and milrinone (0.2 μg/kg/min) was initiated. Gradually, the CVP returned to 12 mmHg, and TEE showed improved LV and RV motions, with rightward shift of the intraventricular septum. At the beginning of OLV, the pressure-controlled ventilation mode was adjusted; peak inspiratory pressure and positive end-expiratory pressure were changed from 17 cmH_2_O to 28 cmH_2_O and from 5 cmH_2_O to 7 cmH_2_O, respectively, to maintain saturation of percutaneous oxygen (SpO_2_) above 90%. After insertion of the access ports, SpO_2_ decreased to 91% at FiO_2_ of 1.0, pump power consumption increased to 3.5 watts, and the CVP reached 20 mmHg, when the mean ABP was maintained at approximately 85 mmHg (Fig. 4 (B)). TEE showed severely reduced RV motion, and the intraventricular septum had again shifted slightly to the left side. The RVSP was calculated at 35 mmHg, whereas the RV-FAC was calculated at 17.7% and appeared to be near the pre-OLV level. For further support of right heart function, continuous intravenous infusion of milrinone was increased to 0.4 μg/kg/min. Arterial blood gas analysis at this time revealed the following results: pH 7.357, pCO2 42.9 mmHg, pO2 67.9 mmHg, BEecf − 1.4, lactate 0.6 mmol/L, SaO2 96.7% and Hb 10.2 g/dL at FiO2 1.0. After a couple of min, TEE examination showed that the LV and RV motions had slightly improved, and the intraventricular septum had shifted toward the RV. Reflecting gradually improved RV wall motion, the RV-FAC was found to be 33.8%. With the increase in urine output, oxygenation improved and CVP decreased to 10 mmHg. The TEE findings did not change, and the mean ABP was maintained at 64–85 mmHg. The pulsatile waveforms were maintained on ABP and SpO_2_ throughout the operation. Intraoperatively, the EVAHEART® monitor showed that the pump speed was set at 1700 rpm; the pump power consumption was 2–4 W. The VATS was completed successfully with stable hemodynamic conditions.

Intraoperatively, the total administration of fentanyl was 450 μg. For postoperative pain control, a single intercostal nerve block (ICNB) was applied by the thoracic surgeons from the inside whilst closing the chest, and an intravenous patient control system (fentanyl 20 μg/h, lock-out time 10 min, 20 μg/one demanded dose) was used. The total OLV time was 3 h 6 min, with an operation time of 3 h 5 min, necessitating a fluid input of 1320 ml crystalloids. The total urine output and blood loss measured were 2550 ml and 23 ml, respectively. Before recovery from general anesthesia, the TEE findings and LVAD parameters showed unremarkable changes in the supine position. The neuromuscular blockade was reversed with sugammadex sodium. After extubation, the patient did not experience pain and could breathe deeply. The patient was transferred to the intensive care unit in stable condition. However, he needed temporary bilevel positive airway pressure support after several hours. Moreover, postoperative pneumonia was suspected on postoperative day 2, and a change in the antibiotic protocol was needed. Finally, he recovered in 1 week and was discharged from our hospital in stable condition on postoperative day 37.

## Discussion and conclusions

For patients with LVAD, the right heart function is frequently marginal; hence, it is important to avoid factors that worsen PVR (e.g., hypoxia, hypercarbia, and light anesthesia) and overfilling the RV with fluid [11–13]. However, OLV could lead to higher PVR, induced by possible complications such as hypoxemia and hypercarbia, where the RV encounters sudden changes in afterload, preload, and contractility [14]. Therefore, based on the findings of previous studies [3–10], the management of LVAD-supported patients for VATS may be more challenging than that of LVAD-supported patients undergoing noncardiac surgeries, as there may be sudden OLV-associated, deteriorative RV function.

Generally, medical therapy for acute right heart failure starts with meticulous volume optimization. Subsequent management strategies involve inotropes to reduce the cardiac filling pressures (dobutamine and phosphodiesterase III inhibitors) and further measures for afterload reduction [inhaled nitric oxide (iNO)] [15, 16]. NO is administered as a pharmacological intervention to treat right heart failure caused by pulmonary hypertension [15, 16]. However, it remains controversial whether iNO is effective in hypoxia during OLV. Therefore, routine use of iNO to treat hypoxemia during OLV is not recommended [17, 18].

In our present case, relatively preserved right heart function (trivial TR and low CVP and RVSP) could tolerate an increase in PVR caused by OLV-induced hypoxia with administration of dobutamine and milrinone and/or adjustment of the ventilator.

In fluid therapy of a patient with LVAD undergoing noncardiac surgery, hypovolemia can compromise not only the perfusion of vital organs but also LVAD performance, although excessive fluid administration may lead to overload in the interstitial space, with increased pulmonary complications, delayed recovery, hypoxia, induced pulmonary hypertension, and right heart failure [12].

After LVAD implantation, the patient’s clinical course was hemodynamically stable. However, it was difficult to maintain the body weight constant due to excessive urination caused by diuretic administration and high or low intake of water, salt, and food. The body weight was controlled by discontinuing the administration of diuretics and adjusting the intake of food, salt, and water. However, pneumothorax recurred, and the body weight was decreased by loss of appetite. Consequently, the patient lost body weight preoperatively, and needed volume load to avoid occurrence of the sucking phenomenon and thrombus formation.

According to Shah et al., at left ventricular internal diastolic dimension (LVIDD) 7 cm, the hazard ratio for axial configuration device thrombosis, compared with the centrifugal configuration device, was 1.61 and continued to rise as the LVIDD increased. It was considered that the patient potentially had higher thrombotic risk because the preoperative LVIDD of the patient was measured at 7.7 cm, although EVAHEART® is a centrifugal LVAD. Conversely, in multivariable models, the hazard of stroke was higher with the CC device regardless of LVIDD [19]. In addition, interruption of anticoagulation due to the bleeding risk associated with an elective procedure may also lead to an elevated risk of thromboembolism, even if heparin bridging is implemented. A recent study recommended an extended personalized approach that incorporates the extent of the patient’s underlying risk for thrombosis and bleeding [20].

In LVAD patients, severe right heart failure is a thrombotic risk factor [20] and may be induced by hypovolemia. Therefore, preoperative volume management with anticoagulation therapy is important to avoid thrombus formation.

Consequently, the patient had been hydrated with crystalloids, and a weight gain of 3.5 kg in 4 days was achieved before the VATS, exceeding his target body weight by 1 kg, as the optimal volume management to compensate for body weight loss. Hemodynamic stability was achieved at anesthesia induction, although we had to manage deteriorative desaturation during OLV. Finally, the total fluid balance was minus 1253 ml, and oxygenation during OLV gradually improved, accompanied with an increase in the volume of urine, which was drained continuously.

EVAHEART®, i.e., the patient’s LVAD, acts as an implantable centrifugal blood pump. It is used for bridge-to-transplantation and has a gentle-slope pressure-flow characteristic and an extremely high pump flow capacity of 15–20 l/min during systole [21]. Continuous-flow LVADs generate flow that may present pulsatility because of the residual LV function of the assisted heart. Indeed, the presence of pulsatility on pump signals has been used as an anecdotal “marker” of the myocardial contractile state in the clinical realm [11, 21–24]. Therefore, it is anticipated that the pump flow for patient management does not need to be known if the pulsatility is maintained on an arterial line and/or SpO_2._ Intraoperatively, in the present case, the pump flow pattern was completely pulsatile at a constant speed of 1700 rpm, with a stable pump power consumption at 2–4 W.

Monitoring of CVP and pulmonary artery catheter (PAC) placement should be considered on a case-by-case basis [4, 12, 13]. In this case, we considered that a PAC was not necessary because the patient showed no evidence of pulmonary hypertension and his right heart function was sufficiently preserved to resist the increase in PVR associated with OLV. In addition, TEE was anticipated to replace PAC because TEE can provide quantification of pulmonary artery systolic pressure by the trans-tricuspid pressure gradient, which is a reliable method compared to PAC [15]. Practically, in conjunction with CVP, TEE was useful to assess the deteriorative hemodynamic changes following OLV and change in patient position and to guide fluid therapy and inotropic drug administration. However, PAC should be considered in cases of pulmonary hypertension and/or highly predictable hemodynamic instability during surgery because of progressive right heart failure, for example, for a LVAD patient with low PAP and high CVP [13] .

A change to the lateral decubitus position could also possibly affect LVAD performance because of decrease in preload [3, 12, 13]. In the current case, we suspected insufficient anesthesia, as a change of the patient position to the lateral decubitus position resulted in a transient increase in PVR. In addition to rocuronium bromide and remifentanil, dobutamine and milrinone were administered to improve right heart function and LVAD performance. Eventually, hemodynamic stability was achieved.

Regarding postoperative analgesia, there is no gold standard for regional analgesia for VATS. In contrast, thoracic epidural analgesia and paravertebral block are established analgesic gold standards for open surgery such as thoracotomy [25]. Recently, several types of regional analgesia have been reported for VATS such as multilevel and single-shot paravertebral blocks, interpleural infusion, long thoracic nerve block [25], retrolaminar block, erector spinae plane block [26], and continuous and single-shot serratus plane blocks [27]. These procedures may be applied even for LVAD patients who require anticoagulation. However, no study has examined the frequency and severity of hemorrhagic complications after plexus or peripheral block in patients receiving anticoagulation therapy. For patients undergoing deep plexus or deep peripheral block, recommendations regarding neuraxial techniques should be similarity applied [28].

In our case, for postoperative pain control, intravenous patient-controlled analgesia with fentanyl was used and a single shot of ICNB was applied by the thoracic surgeons before closing the chest; this was favored due to the lower incidence of serious adverse events such as epidural hematoma, despite poorer pain control than that after thoracic epidural anesthesia [29].

The type of anesthesia (inhalational versus total intravenous anesthesia) by itself does not affect oxygenation during OLV [14, 17], although intravenous anesthetic drugs have a limited effect on hypoxic pulmonary vasoconstriction, which is attenuated by inhaled anesthetics [30]. Because the patient was diagnosed with Becker type muscular dystrophy, we chose total intravenous anesthesia to avoid the potential risk of malignant hyperthermia, although controversial results have been reported regarding whether malignant hyperthermia is caused by administration of succinylcholine rather than volatile anesthetic agents [31, 32]. We believe that it is not crucial to select the type of anesthesia in LVAD patients that require OLV.

The anesthetic management of a patient with LVAD in VATS is challenging because the possible hemodynamic changes induced by hypoxia associated with OLV affect LVAD performance and right heart function.

In our experience, VATS that requires OLV will be well tolerated in a patient with LVAD with preserved right heart function, and a multidisciplinary approach to maintain right heart function will be needed.

## Data Availability

The datasets used or presented during this study are available from the corresponding author on request.

## Abbreviations

ABP: Arterial blood pressure
APTT: Activated partial thromboplastin time
CT: Computed tomography
CVP: Central venous pressure
FAC: Fractional area change
ICNB: Intercostal nerve block
iNO: inhaled nitric oxide
LV: Left ventricle
LVAD: Left ventricular assist device
LVIDD: Left ventricular internal diastolic dimension
OLV: One-lung ventilation
PAC: Pulmonary artery catheter
PT-INR: Prothrombin time-international normalized ratio
PVR: Pulmonary vascular resistance
RV: Right ventricle
RVSP: Right ventricular systolic pressure
SpO2: Saturation of percutaneous oxygen
TAP: Tricuspid annuloplasty
TEE: Transesophageal echocardiography
TR: Tricuspid regurgitation
VATS: Video-assisted thoracic surgery

## References

[1] Nelson EW, Heinke T, Finley A, Guldan GJ, Gaddy P, Toole JM (2015). Management of LVAD patients for noncardiac surgery: a single-institution study. J Cardiothorac Vasc Anesth.

[2] Mathis MR, Sathishkumar S, Kheterpal S, Caldwell MD, Pagani FD, Jewell ES (2017). Complications, risk factors, and staffing patterns for noncardiac surgery in patients with left ventricular assist devices. Anesthesiology.

[3] Goldstein DJ, Mullis SL, Delphin ES, el-Amir N, Ashton RC, Gardocki M (1995). Noncardiac surgery in long-term implantable left ventricular assist-device recipients. Ann Surg.

[4] Stone ME, Soong W, Krol M, Reich DL (2002). The anesthetic considerations in patients with ventricular assist devices presenting for noncardiac surgery: a review of eight cases. Anesth Analg.

[5] Oleyar M, Stone M, Neustein SM (2010). Perioperative management of a patient with a nonpulsatile left ventricular-assist device presenting for noncardiac surgery. J Cardiothorac Vasc Anesth.

[6] Ficke DJ, Lee J, Chaney MA, Bas H, Vidal-Melo MF, Stone ME (2010). Case 6-2010: noncardiac surgery in patients with a left ventricular assist device. J Cardiothorac Vasc Anesth.

[7] Kartha V, Gomez W, Wu B, Tremper K (2008). Laparoscopic cholecystectomy in a patient with an implantable left ventricular assist device. Br J Anaesth.

[8] Chacon MM, Hattrup EA, Shillcutt SK (2014). Perioperative management of two patients with left ventricular assist devices presenting for noncardiac surgery in the prone position. A A Case Rep.

[9] Sathishkumar S, Kodavatiganti R, Plummer S, High K (2012). Perioperative management of a patient with an axial-flow rotary ventricular assist device for laparoscopic ileo-colectomy. J Anaesthesiol Clin Pharmacol.

[10] Nayak JG, White CW, Nates W, Sharda R, Home D, Kaler K (2013). Laparoscopic nephroureterectomy in a patient with a left ventricular assist device. Can Urol Assoc J.

[11] Chung M (2018). Perioperative management of the patient with a left ventricular assist device for noncardiac surgery. Anesth Analg.

[12] Slininger KA, Haddadin AS, Mangi AA (2013). Perioperative management of patients with left ventricular assist devices undergoing noncardiac surgery. J Cardiothorac Vasc Anesth.

[13] Dalia AA, Cronin B, Stone ME, Turner K, Hargrave J, Vidal-Melo MF (2018). Anesthetic management of patients with continuous-flow left ventricular assist devices undergoing noncardiac surgery: an update for anesthesiologists. J Cardiothorac Vasc Anesth.

[14] Rana M, Yusuff H, Zochios V (2019). The right ventricle during selective lung ventilation for thoracic surgery. J Cardiothorac Vasc Anesth.

[15] Harjola VP, Mebazaa A, Čelutkienė J, Bettex D, Bueno H, Chioncel O (2016). Contemporary management of acute right ventricular failure: a statement from the heart failure association and the working group on pulmonary circulation and right ventricular function of the European society of cardiology. Eur J Heart Fail.

[16] Lin W, Poh AL, Tang WHW (2018). Novel insights and treatment strategies for right heart failure. Curr Heart Fail Rep.

[17] Karzai W, Schwarzkopf K (2009). Hypoxemia during one-lung ventilation: prediction, prevention, and treatment. Anesthesiology.

[18] Campos JH, Feider A (2018). Hypoxia during one-lung ventilation: a review and update. J Cardiothorac Vasc Anesth.

[19] Shah P, Birk S, Maltais S, Stulak J, Elmi A, Pagani FD (2017). Left ventricular assist device outcomes based on flow configuration and pre-operative left ventricular dimension: an interagency registry for mechanically assisted circulatory support analysis. J Heart Lung Transplant.

[20] Shah P, Tantry US, Bliden KP, Gurbel PA (2017). Bleeding and thrombosis associated with ventricular assist device therapy. J Heart Lung Transplant.

[21] Yamazaki K, Saito S, Kihara S, Tagusari O, Kurosawa H (2007). Completely pulsatile high flow circulatory support with a constant-speed centrifugal blood pump: mechanisms and early clinical observations. Gen Thorac Cardiovasc Surg.

[22] Ferreira AL, Wang Y, Gorcsan J, Antaki JF (2011). Assessment of cardiac function during mechanical circulatory support: the quest for a suitable clinical index. Conf Proc IEEE Eng Med Biol Soc.

[23] Moazami N, Fukamachi K, Kobayashi M, Smedira NG, Hoercher KJ, Massiello A (2013). Axial and centrifugal continuous-flow rotary pumps: a translation from pump mechanics to clinical practice. J Heart Lung Transplant.

[24] Lim HS, Howell N, Ranasinghe A (2017). The physiology of continuous-flow left ventricular assist devices. J Card Fail.

[25] Steinthorsdottir KJ, Wildgaard L, Hansen HJ, Petersen RH, Wildgaard K (2014). Regional analgesia for video-assisted thoracic surgery: a systematic review. Eur J Cardiothorac Surg.

[26] Onishi E, Toda N, Kameyama Y, Yamauchi M (2019). Comparison of clinical efficacy and anatomical investigation between retrolaminar block and erector spinae plane block. Biomed Res Int.

[27] Allain PA, Carella M, Agrafiotis AC, Burey J, Assouad J, Hafiani EM (2019). Comparison of several methods for pain management after video-assisted thoracic surgery for pneumothorax: an observational study. BMC Anesthesiol.

[28] Horlocker TT (2011). Regional anaesthesia in the patient receiving antithrombotic and antiplatelet therapy. Br J Aaesth.

[29] Umari M, Falini S, Segat M, Zuliani M, Crisman M, Comuzzi L (2018). Anesthesia and fast-track in video-assisted thoracic surgery (VATS): from evidence to practice. J Thorac Dis.

[30] Lumb AB, Slinger P (2015). Hypoxic pulmonary vasoconstriction: physiology and anesthetic implications. Anesthesiology.

[31] Cripe LH, Tobias JD (2013). Cardiac considerations in the operative management of the patient with Duchenne or Becker muscular dystrophy. Paediatr Anaesth.

[32] Segura LG, Lorenz JD, Weingarten TN, Scavonetto F, Bojanić K, Selcen D (2013). Anesthesia and Duchenne or Becker muscular dystrophy: review of 117 anesthetic exposures. Paediatr Anaesth.

